# How I do it: endoscopic endonasal resection of tuberculum sellae meningioma

**DOI:** 10.1007/s00701-021-04784-5

**Published:** 2021-03-05

**Authors:** Markus Wiedmann, Aslan Lashkarivand, Jon Berg-Johnsen, Daniel Dahlberg

**Affiliations:** 1 Department of Neurosurgery, Oslo University Hospital, PO box 4950 Nydalen, 0424 Oslo, Norway; 2 Institute of Clinical Medicine, Faculty of Medicine, University of Oslo, Oslo, Norway

**Keywords:** Skull base surgery, Endoscopic endonasal approach, Meningioma, Microsurgical technique, Visual evoked potentials

## Abstract

**Background:**

Tuberculum sellae meningiomas (TSMs) adherent to neurovascular structures are particularly challenging lesions requiring delicate and precise microneurosurgery. There is an ongoing debate about the optimal surgical approach.

**Method:**

We describe technical nuances and challenges in TSM resection using the endoscopic endonasal approach (EEA) in two cases of fibrous tumors with adherence to neurovascular structures. The cases are illustrated with a video (case 1) and figures (cases 1 and 2).

**Conclusion:**

A dedicated team approach and precise microsurgical technique facilitate safe resection of complex TSMs through the EEA.

**Supplementary Information:**

The online version contains supplementary material available at 10.1007/s00701-021-04784-5.

## Introduction and relevant surgical anatomy

Tuberculum sellae meningiomas (TSMs) arise from the tuberculum sellae, limbus sphenoidale, and chiasmatic sulcus and tend to infiltrate the medial aspect of one or both optic canals [4]. This area is accessed with superb visualization through the endoscopic endonasal approach (EEA) [2]. Advantages of the EEA are early exposure and devascularization of the tumor base, decompression of the optic nerves from medial without brain or nerve retraction, and clear visualization of perforators to the optic apparatus and pituitary stalk. However, there seems to be a tendency to treat complex TSMs with transcranial approaches [3]. We illustrate the technical nuances on how to approach complex TSMs using the EEA without compromising on microsurgical technique.

Understanding the anatomy of the nasal cavity and paranasal sinuses is paramount for a safe and efficient endonasal approach to the anterior skull base. We carefully evaluate endonasal anatomy preoperatively, and in cases where the EEA is unsuitable, either due to pathology or significant anatomical variation, a transcranial approach is preferred. The tuberculum is laterally bordered by the tubercular crest, represented by the medial optico-carotid recess (mOCR) as seen from the EEA. The course of the optic nerves runs in the cisternal, preforaminal, intracanalicular, and intraorbital segments. The lateral optico-carotid recess corresponds to the optic strut and is a landmark for the intracanalicular segment. The dural fold of the limbus sphenoidale forms the falciform ligament as it extends laterally [1]. Small perforators and the superior hypophyseal artery (SHA) arise from the medial wall of the internal carotid artery (ICA) supplying the optic apparatus and the pituitary gland.

## Description of the technique

**Fig. 1:**
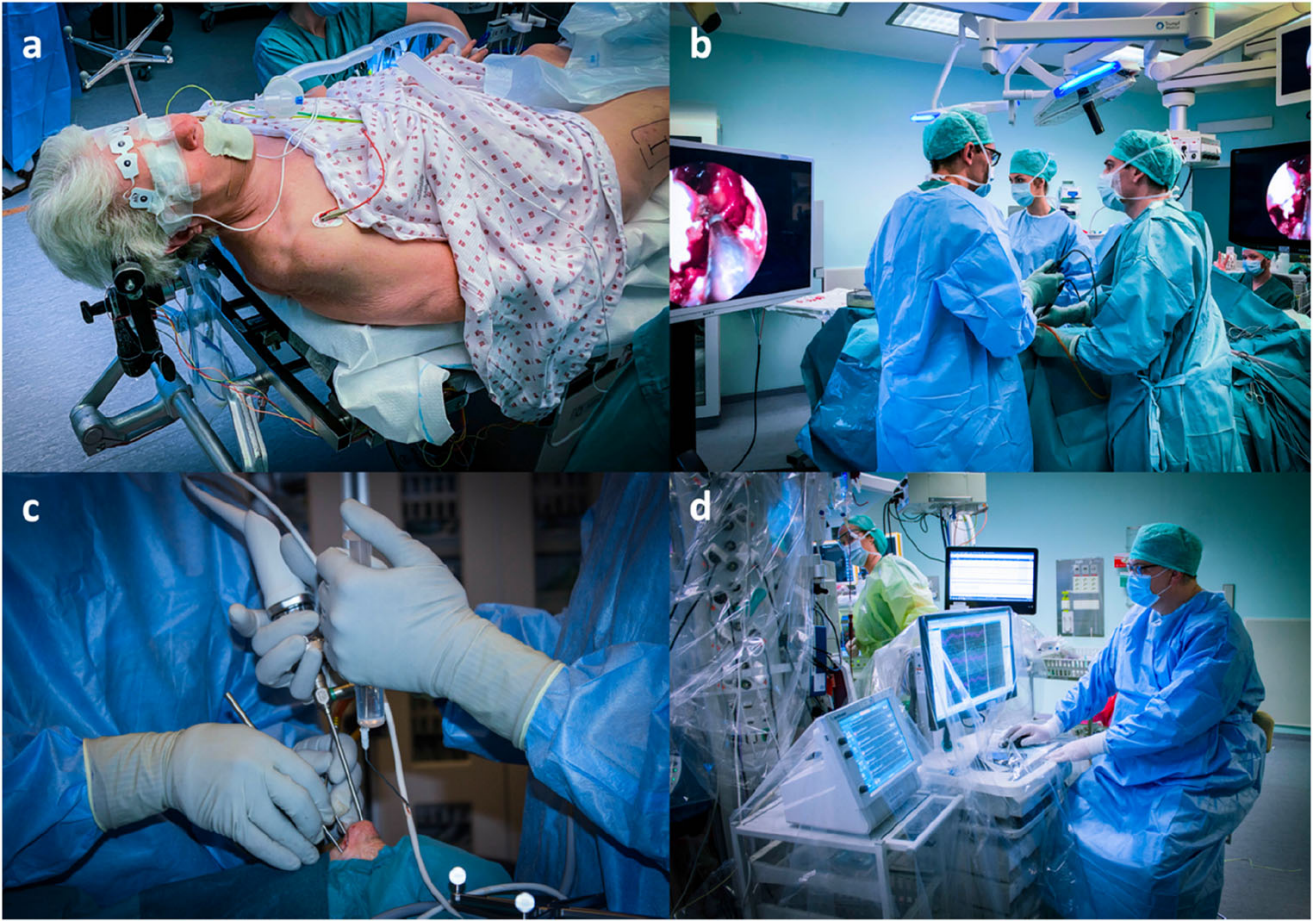
Patient and surgeon position. **a** Head fixed, elevated, 15° turned to the right and slightly laterally flexed to the left to preserve venous drainage and improve surgeon ergonomics. **b** Comfortable working position for both surgeons, slightly facing each other and their separate screens. **c** Bimanual neurosurgical technique using both nostrils. The hands are in a naturally relaxed position. **d** Monitoring visual evoked potentials during surgery at the foot end of the patient

Surgery is performed via a binostril 4-hand approach. The surgeons are standing slightly opposite, facing two different screens (Fig. 1), thus increasing the freedom of movements, ergonomics, and communication. The main authors (MW, DD) have established a routine of reversing roles every 30 min, where one surgeon primarily dissects, while the other provides visualization and assistance. This has increased workflow and efficiency, while reducing fatigue during long-lasting surgeries.

A nasoseptal flap is harvested and placed in the nasopharynx. Septoplasty, bilateral uncinectomy with maxillary antrostomy, resection of the ethmoid bullae, and posterior ethmoidectomies are performed according to the endonasal anatomy for unhindered access. The middle turbinates are displaced laterally in the space created by this maneuver and can normally be spared. This is followed by a partial superior turbinectomy, a wide sphenoidotomy, posterior nasal septectomy, and removal of the sphenoid rostrum (Video).

Bone overlying the sella, parasellar ICAs, and planum is egg-shelled with a 4-mm coarse diamond burr and dissected off the dura. The tubercular strut is thinned, divided, and removed in pieces. Its lateral extension, the mOCR, corresponds to the preforaminal segment of the optic nerve and needs to be removed completely, together with the middle clinoid process, lying just inferiorly to the mOCR.

If there is evidence of tumor growth into the optic canal on preoperative MRI, the orbital apex and mOCR are exposed before decompressing the optic canal.

**Fig. 2:**
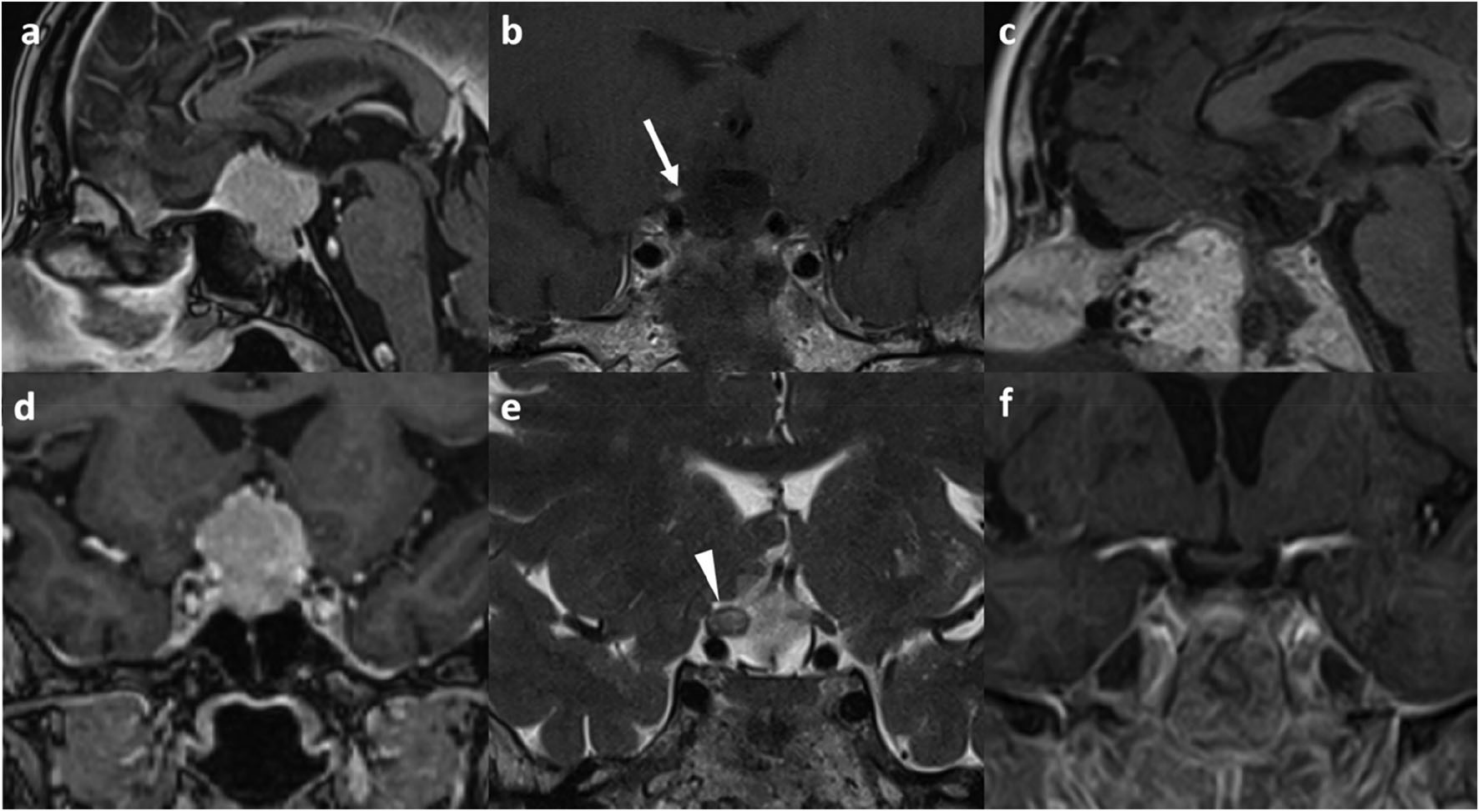
Tuberculum sellae meningioma WHO grade I (*case 1*). **a**, **d** Contrast-enhanced MRI. There was invasive tumor growth into the right optic nerve (see Video). **b** Contrast-enhanced T1-weighted image shows enhancement in the right optic nerve (arrow) and **e** optic nerve swelling on T2-weighted image (arrowhead). Otherwise, there was no tumor residual (**c–f**). Pituitary function was preserved. Initially, the patient presented with acute onset visual decline on the right eye and hemianopia. Visual function of the right eye was marginal and could not be preserved. Vision improved immediately on the left eye

Once bony exposure is completed; the dural base of the tumor is devascularized. Dural opening starts with a vertical midline incision from the anterior tumor border to the upper border of the pituitary gland. Then, a horizontal cut, guided by neuronavigation, is made just in front of the anterior margin of the tumor (T incision). We believe this maneuver is safer than performing two laterally curved incisions just medial to the optic canals, as the remaining dural flaps involved with tumor can be gradually reflected and cut safely. Debulking of fibrous tumors is performed using ultrasonic aspiration and microsurgical piecemeal resection. Establishing arachnoidal dissection planes early and identifying areas of arachnoidal breach with adherence to, or encasement of, neurovascular structures is important for safe and efficient tumor removal (Fig. 2).

**Fig. 3:**
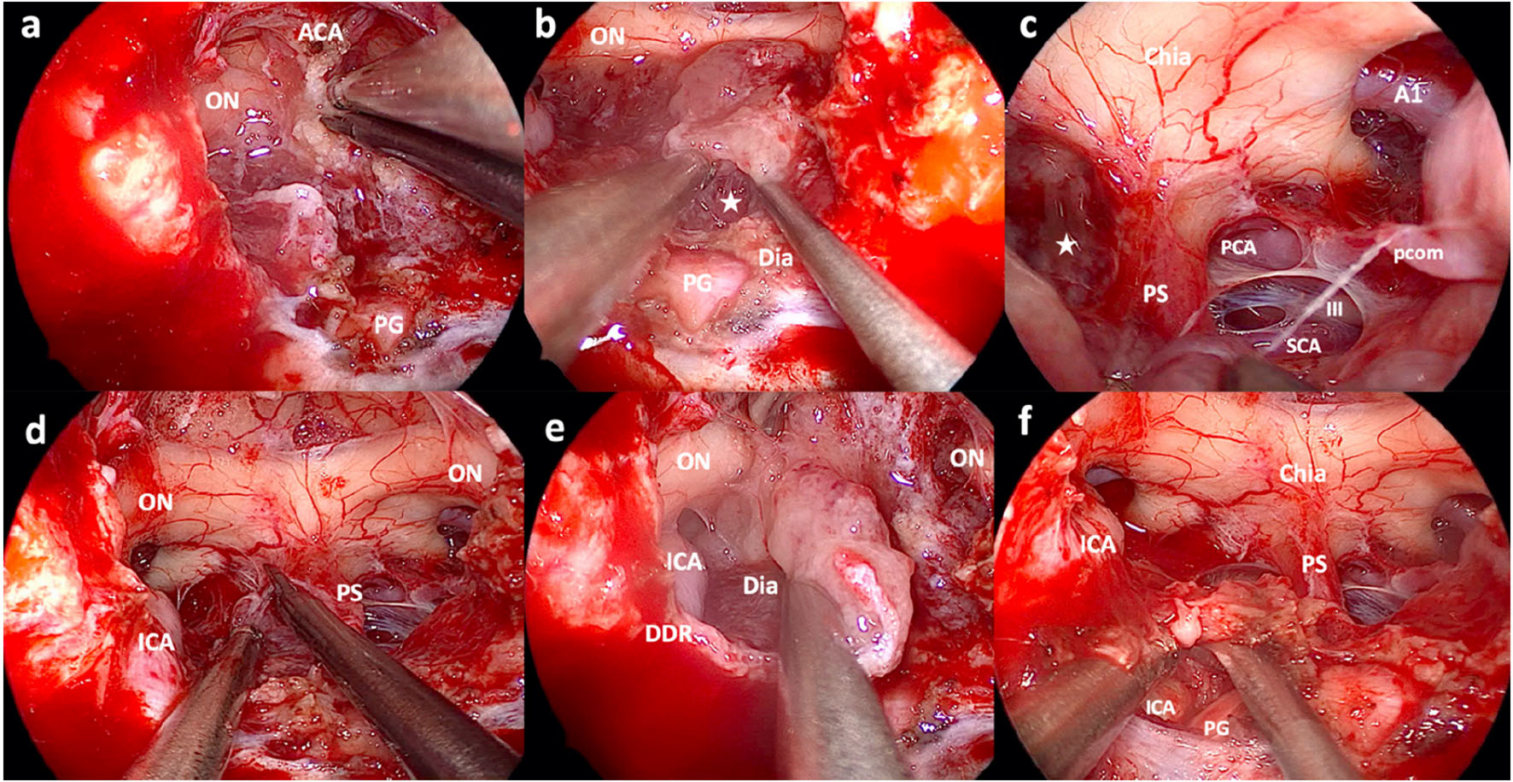
Intraoperative photos of tumor resection in *case 2*. **a** Sharp dissection of the adherent tumor from the anterior cerebral artery complex. **b** After opening the diaphragm, the PS (asterisk) is identified by dissecting along the surface of the PG. **c** Panoramic view after tumor resection from the PS, chiasm, and PCOM artery. There still is remaining tumor to the patient`s right (asterisk). **d** Sharp dissection of tumor from the PS and chiasm while preserving microvasculature. **e** Diaphragm covering cavernous tumor component. This is sharply opened for tumor resection (see next photo). **f** Tumor is dissected from the PG medially and the cavernous part of the ICA laterally. Bleeding from the cavernous sinus is controlled by injection of a hemostatic matrix with thrombin (ON = optic nerve; ACA = anterior cerebral artery complex; PG = pituitary gland; Dia = diaphragm; Chia = optic chiasm; PS = pituitary stalk; A1 = A1 segment of anterior cerebral artery; PCA = posterior cerebral artery; SCA = superior cerebellar artery; III = oculomotor nerve; DDR = distal dural ring; PCOM = posterior communicating artery; ICA = internal carotid artery)

We establish a dissection plane anterior to the rostral tumor margin at the planum and identify the A2 segments. Tumor debulking is performed as we proceed to the inferior margin where tumor is separated from the pituitary gland and stalk. Often, the sellar diaphragm has to be resected to identify and preserve the pituitary stalk (Fig. 3). Tumor is mobilized and separated from the chiasm and optic nerves. Sharp dissection is performed to minimize the risk of injury to neurovascular structures and perforators supplying the optic apparatus (Fig. 3 and Video). The optic nerves are followed to the optic canals where preforaminal, and if present, intracanalicular, tumor is resected.

**Fig. 4:**
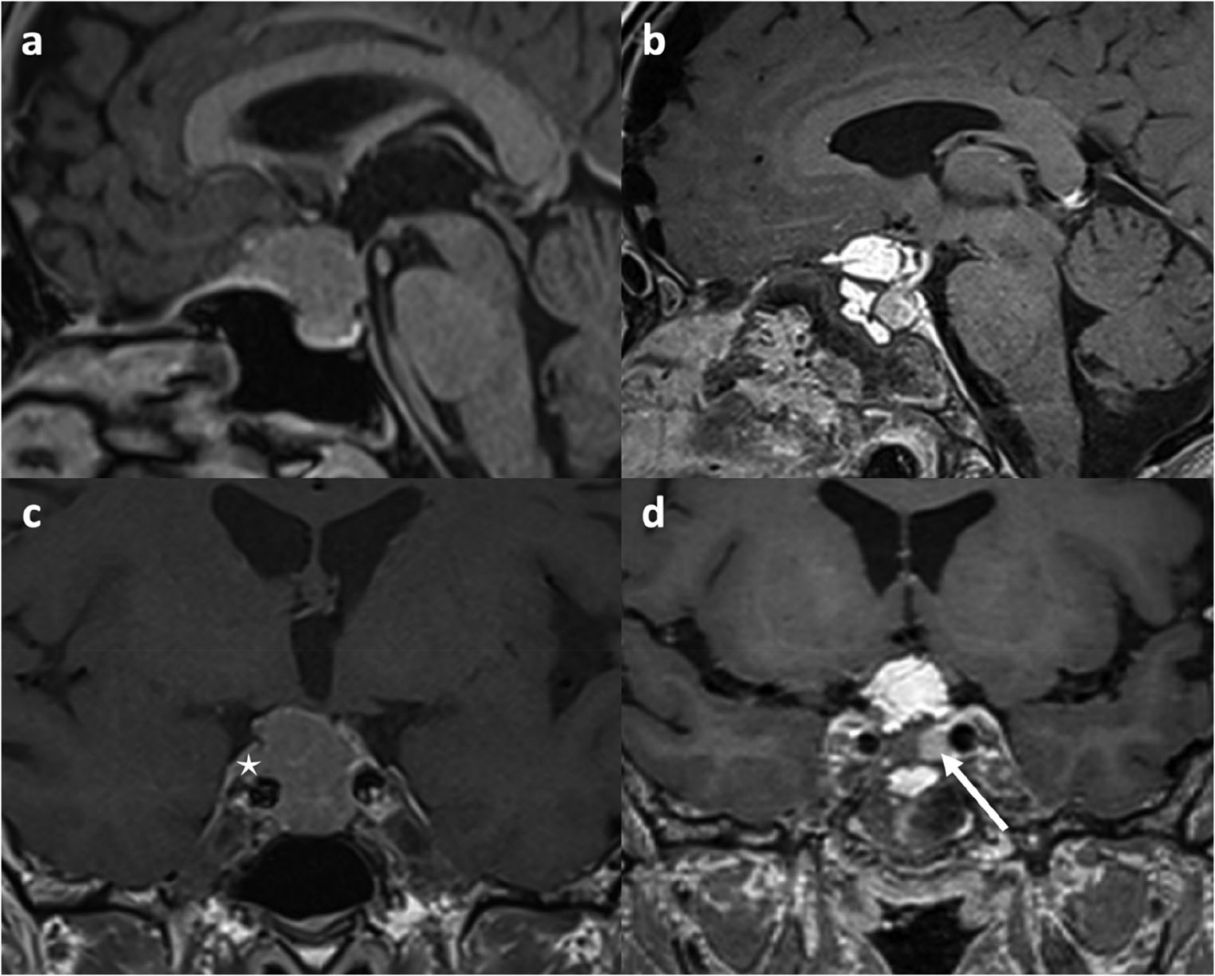
Tuberculum sellae meningioma WHO grade I (*case 2*). **a**–**c** Preoperative contrast-enhanced MRI shows a sellar and suprasellar tumor with invasion of the right cavernous sinus (asterisk). **b**, **d** Postoperative contrast-enhanced MRI demonstrating gross total resection and fat graft in the tumor cavity. The pituitary gland was displaced to the patient’s left (white arrow). Vision and visual fields improved rapidly after surgery and pituitary function was preserved

The skull base defect requires meticulous reconstruction. We use collagen matrix (Duragen Plus, IntegraLifesciences) inlay and fascia lata onlay, covered by a vascularized nasoseptal flap. A fat graft is sometimes used for filling dead space (Fig. 4). We do not routinely use a lumbar drain and patients are mobilized the day after surgery [5].

## Indications

TSMs without significant tumor growth lateral to the carotid arteries. Redo surgery and tumor involvement of vascular structures or the optic apparatus are not contraindications to the EEA, but require a skilled endoscopic skull base team.

## Limitations

Tumor encasing or extending lateral to the optic nerves.

## Specific perioperative considerations

Preoperative:

- Brain MRI with fat-suppression contrast-enhanced T1 and thin T2/CISS sequences, CT of the skull base and paranasal sinuses, CT angiogram, neuro-ophthalmological evaluation, and endocrine assessment

Intraoperative:

- Neuronavigation, micro-Doppler, and visual evoked potentials (VEP)

Postoperative:

- Sphenoidal packing and nasal splint for 10–14 days
- Routine 3-month follow-up with MRI, endocrine, and neuro-ophthalmologic assessment
- If a CSF leak occurs (verified by beta-trace protein test), we re-operate with exploration and re-adjustment of the skull base reconstruction

## How to avoid complications

- Assessment of preoperative imaging for optic nerve displacement, lateral tumor extension, invasion of optic canals, breach of arachnoid planes, adhesion to neurovascular structures, and position of the pituitary gland and stalk.
- Comorbidities, such as diabetes mellitus, smoking, chronic sinusitis, and previous sinus surgery, increase the risk of postoperative cerebrospinal fluid (CSF) leakage and infection. These issues must be addressed and optimized pre- and postoperatively.
- Preservation of the microvasculature to the optic apparatus is key to avoid visual deterioration.

## Specific information to give to the patient about surgery and potential risks

Surgical risks related to anterior skull base surgery and EEA, in particular, visual and olfactory deterioration, endocrine dysfunction, and CSF leakage.

## A summary of 10 key points

1. Operating room setup and patient positioning are important for optimal workflow, communication, and ergonomics during surgery.
2. A well-concerted surgical team is the key to success.
3. Bimanual microsurgical technique is necessary for safe and efficient surgery.
4. VEP monitoring may contribute to visual preservation.
5. A wide exposure with removal of the mOCRs, the bone overlying the ICAs, and adequate opening of the planum facilitates tumor access.
6. Tumor extension into the optic canal is not a limitation for achieving gross total resection with the EEA.
7. Removal of the endoscope irrigation channel reduces its diameter and limits “sword fights” during microsurgical resection.
8. Care is taken to preserve small perforators to the optic apparatus.
9. In case of infiltrative growth or significant adherence to neurovascular structures, radical resection may lead to unacceptably high morbidity.
10. Watertight multilayered reconstruction with a vascularized nasoseptal flap is routinely performed.

## Supplementary Information

Video 1 Endoscopic endonasal resection of a fibrous tuberculum sellae meningioma with invasive growth into the right optic nerve (MP4 242531 kb)
